# The effect of cumulative exposure with unhealthy lifestyles on the H-type hypertension among Chinese adults: a community-based, propensity-score-matched, and case–control study

**DOI:** 10.3389/fnut.2024.1470788

**Published:** 2024-09-18

**Authors:** Ling Li, Jia Wang, Jing Li, Minqi Li, Tianyao Long, Yangyi Zhengliu, Yuan Lv, Xiuqin Hong

**Affiliations:** ^1^Clinical Epidemiology Research Office, Hunan Provincial People's Hospital (The First Affiliated Hospital of Hunan Normal University), Changsha, China; ^2^Cerebral Vascular Disease Rehabilitation Clinical Research Center, Hunan Provincial People's Hospital (The First Affiliated Hospital of Hunan Normal University), Changsha, China; ^3^Department of Scientific Research, Hunan Provincial People's Hospital (The First Affiliated Hospital of Hunan Normal University), Changsha, China; ^4^Key Laboratory of Molecular Epidemiology, Hunan Normal University, Changsha, China

**Keywords:** cumulative exposure effect, unhealthy lifestyles, H-type hypertension (HTH), Chinese community population, propensity score matching, case–control study

## Abstract

**Objective:**

To assess whether cumulative exposure of unhealthy lifestyles is associated with HTH in Chinese adults and to explore the combination of unhealthy lifestyles.

**Methods:**

This study combined a community-based cross-sectional study with a 1:1 matched case–control study using propensity scores among adults in six randomly selected districts from Hunan Province, China. We recruited 5,258 people, of whom 4,012 met the criteria. Lifestyles and personal characteristics were collected by a questionnaire. Lifestyle score was calculated using cigarette smoking, heavy alcohol consumption, inactive exercise, unhealthy diet and abnormal BMI. HTH was defined as having a diagnosis of essential hypertension with Hcy ≥ 15 umol/L. Logistic regression models and multivariate analyses were used to explore the associations. We calculated odds ratios (ORs) and attributable risk proportion (ARP) for the association of HTH with lifestyle score. The dose–response relationship was evaluated using restricted cubic splines method.

**Results:**

Of the 4,012 adults, 793 had HTH, with a population prevalence of 19.8%. In the propensity-score-matched case–control study, 1,228 (614 cases and 614 controls) were included, and those with at least four unhealthy lifestyle factors had a higher risk of HTH than those with 0 unhealthy lifestyle factor (adjusted OR = 2.60, 95%CI:1.42–4.78), with an ARP of the cumulative exposure of unhealthy lifestyle was 28.23% (95% CI: 6.34–37.86%). For three unhealthy lifestyles group, the combination of heavy alcohol consumption, unhealthy diet and BMI ≥24 Kg/m^2^ was most associated with HTH (OR = 7.49, 95%CI: 1.12–50.08). For four unhealthy lifestyles group, the combination of smoking, heavy alcohol consumption, unhealthy diet and BMI ≥24 Kg/m^2^ had the greatest correlation with HTH (OR = 3.75, 95%CI: 1.24–7.38). Notably, there was a monotonically increasing curve (J-shaped) relationship between unhealthy lifestyles and the risk of HTH (*p* = 0.014).

**Conclusion:**

Our findings suggest that there was a significant cumulative exposure effect of unhealthy lifestyles on the risk of HTH, with the largest effect combination being heavy alcohol consumption, unhealthy diet and BMI ≥24 Kg/m^2^. Targeted interventions that reducing heavy alcohol consumption, quitting smoking, promoting physical activity and a healthy diet, and keep a normal BMI could substantially reduce the burden of HTH.

## Introduction

Globally, cardiovascular diseases (CVDs) have collectively remained the leading causes of death and substantially caused to loss of health and excess health economic burden (1). Hypertension is the leading and modifiable risk factor for CVD, accounting for one-third of the total global deaths and 1.56 billion adult cases (2). Currently, scientists became interested in new risk factors for CVDs, such as serum homocysteine (Hcy) levels, which has been identified as a potential risk factor for hypertension (3). Previous study revealed that hyper-Hcy (HHcy) were associated with 75% of the hypertension cases (4). In 2008, the concept of H-type hypertension (HTH) proposing essential hypertension combined with HHcy was first introduced by Chinese researchers (5). Notably, the risk of CVDs in patients with HTH was approximately 5 times that of single-hypertension and 12 times that of healthy people (6), and HTH was independently associated with atherosclerotic plaques and stroke (7). Thus, the prevention strategies of HTH are the major global public health issue and challenges today.

Numerous epidemiological studies have established that unhealthy lifestyles, such as long-term heavy drinking, smoking, unhealthy diet, and lack of exercise, May lead to an increase in plasma Hcy levels and ultimately HHcy (8, 9). Detailly, current smoking but not quitting smoking was associated with higher risk of HHcy (10). Additionally, increasing evidence suggests that strict dietary control is highly important for blood pressure control and was one of the means to reduce plasma Hcy levels in hypertensive patient. A diet with rich in fruits and vegetables can lower plasma Hcy levels, thereby reducing the risk of CVD by 7 ~ 9% (11). However, it is worth noting that from the perspective of life course theory (12), there is a cumulative effect of risk factors in the pathogenesis of chronic diseases. Therefore, the study of a single behavioral lifestyle may underestimate its overall risk to health, while the establishment of behavioral lifestyle score can comprehensively reflect the impact of an individual’s cumulative exposure to behavioral lifestyle on population health. For example, behavioral lifestyle score was associated with morbidity and mortality of cancer (13) or cardiovascular diseases (14). However, there was limit study conducted unhealthy lifestyle score so far to assess the its cumulative exposure effect on the HTH. Here, the association between unhealthy lifestyles and HTH will be differed by confounders level (such as age, sex and comorbidity status, etc.) in different directions and magnitudes. Therefore, we conducted a case–control study based on propensity score matching, which adopted a semi-parametric method to increase the possibility of reasonable matching between the case group and the control group, and dealt with multiple confounding factors or stratification, which greatly improved the reliability of the results (15).

Notably, the prevalence of HHcy is much higher among adults in China than in other countries (16–18). Additionally, most of epidemiological studies only focused on hospital-based populations, with few based on general community populations, which May limit interpretation of these data. And, those were mainly concentrated in North China and rare in South China (17). Thus, in this study, we conducted a community-based study design to achieve the following aims: (1) to assess whether cumulative exposure of unhealthy lifestyles is associated with the HTH in Chinese adults; and (2) if such association exists, to explore the combination of unhealthy lifestyles in it.

## Materials and methods

### Study design and population

**Figure 1:**
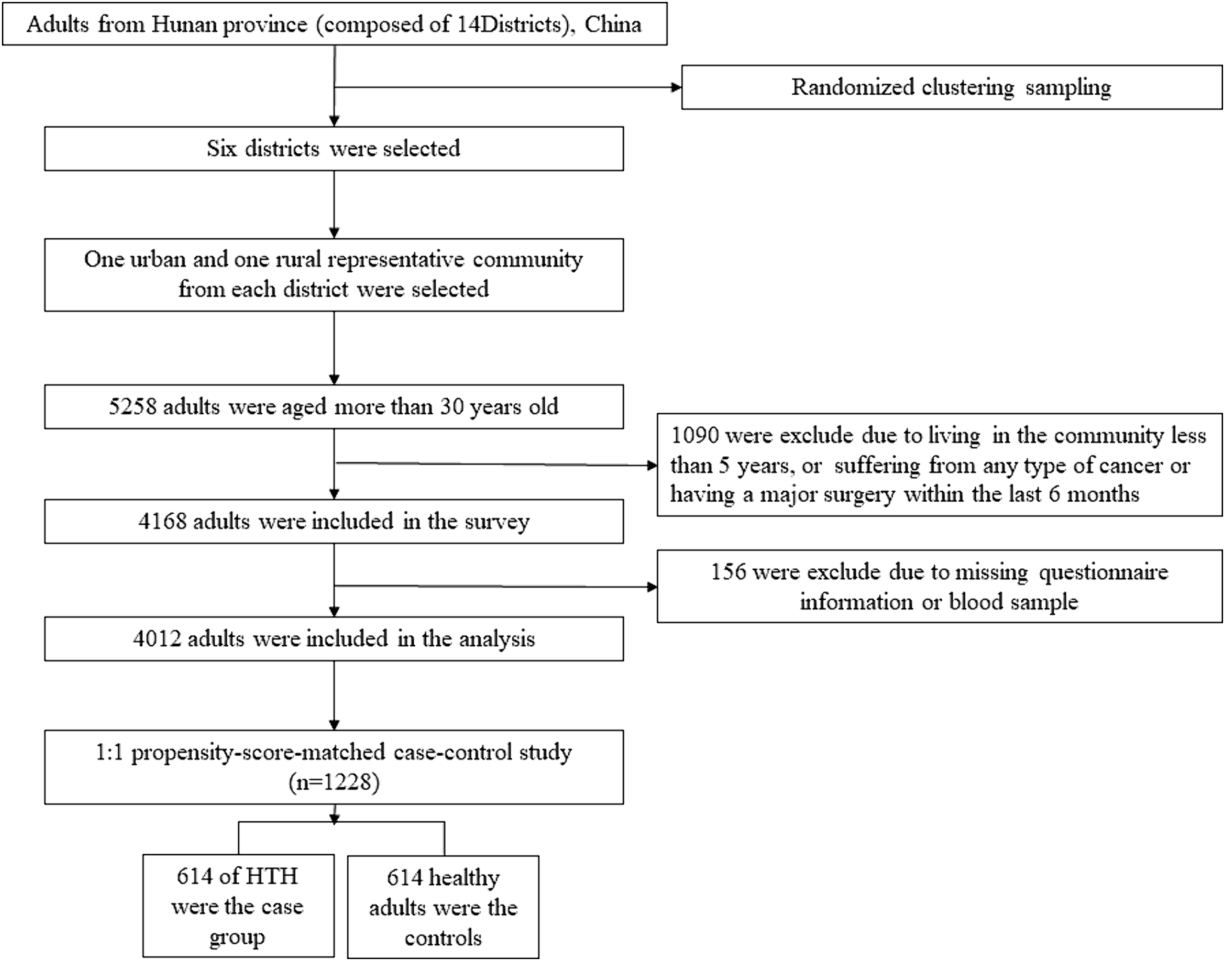
Flowchart of the study.

In this study, we used a study design which combined a population-based cross-sectional study with a 1:1 matched case–control study using propensity scores in Hunan Province (including 14 districts and a population of more than 66 million), China. Detailed information on study design of this population has been described in our previous study (10). Briefly, a multistage cluster random sampling design was carried out to obtain a representative sample from July 2013 to March 2014. First, six districts were randomly selected from the 14 districts. Second, one urban and one rural community were randomly selected from each selected district. Our study included adults over 30 years old, and subjects who lived in the community less than 5 years, suffered from any type of cancer or had a major surgery within the last 6 months were excluded. Those met the inclusion and exclusion criteria signed written informed consent. After obtaining informed consent, eligible participants were asked to complete a questionnaire. Accordingly, 4,012 participants were included in the population-based cross-sectional study to determine the relationship between cumulative exposure and HTH. Furtherly, a 1:1 matched case–control study based on the propensity score matching method (614 healthy participants and 614 HTH) was conducted to verify these cumulative effects (Figure 1).

### Data collection

Demographic characteristics (including age, sex, family income, education, marital status, occupation status and self-reported comorbidities) and lifestyles factor (including smoking, alcohol drinking, exercise and diet) were collected by trained researchers through face-to-face and one-to-one questionnaires, which was referred the questionnaire of the China Health and Retirement Longitudinal Study (CHARLS) (19). A test–retest reliability test was performed on the questionnaire, with a Cronbach’s *α* coefficient in this sample being 0.778. Family income per year was asked for every participant, and further divided into three groups: low, medium and high. Educational attainment was classified as follows: below of high school, ordinary/vocational high school and undergraduate/college degree. Marital status was classified into three groups: unmarried, married/cohabitation and divorce/widow. Occupation included four types: wage-laborer, white-collar worker, farmer and retiree. Self-reported comorbidities included ischemic heart disease, stroke or diabetes.

Height, bodyweight and diastolic/systolic blood pressure were measured by professionally trained nurses using the calibrated electronic automatic tester. Each participant had their blood pressure measured three times with at least 5 min of rest each time. The average of the 3 values was calculated and documented as the final blood pressure value.

Blood samples were collected at 07:30–10:00 after a fasting period of 12 h. The plasma Hcy was measured by trained laboratory technicians using the microplate enzyme immunoassay method, with homocysteine Detection Kit of MedicalSystem Biotechnology Co., Ningbo, China (Reagent batch number, 13082408). Other laboratory indicators included fasting blood-glucose (FPG), plasma total cholesterol (TC), triglyceride (TG), low density lipoprotein cholesterol (LDL-C), high density lipoprotein cholesterol (HDL-C) and C-reactive protein (CRP) were detected using a Hitachi 7,600 Automatic Biochemistry Analyzer (Hitachi).

### Assessment of behavioral lifestyle factors and unhealthy lifestyle score

Smoking was defined in the questionnaire as smoking more than 100 cigarettes in life. Here, a set of simple and easy-to-understand photos to measure the drinks of different kinds of drinks (see in Supplementary Figure S1), and the drink frequency (days) and amounts (drinks) in the past week were reported. The average daily alcohol drinks were estimated as follows: average daily alcohol drinks = (frequency [days] × (amounts [drinks] in each of those days))/7 (10). Heavy alcohol consumption level was defined as average daily alcohol drinks ≥2. For physical activity, the number of days per week that the participants did physical activities (such as weightlifting, stair climbing, fast cycling, aerobics, running, etc.) and the time of each exercise were obtained through the questionnaires. Inactive exercise was defined as having lasting for less than 10 continuous minutes per week (20). We evaluated dietary status using food frequency questionnaire according to a more recent dietary recommendation for blood pressure and combining with traditional Chinese eating habits, which considered adequate consumption of fresh fruit, fresh vegetables, unprocessed meats (including red meat, fish or shellfish), reduced consumption of high-fat, high-salt and sugar-sweetened food. We defined an unhealthy diet as meeting less than four items of the recommendations (see Supplementary methods for details). Body-mass index (BMI) was calculated as bodyweight in kg divided by the square of height in meters, and a value more than or equal to 24 was defined as an abnormal BMI. Additionally, we constructed a composite score of unhealthy lifestyles including cigarette smoking, alcohol consumption, inactive physical exercise, unhealthy diet and abnormal BMI (≥24 Kg/m^2^) on the basis of earlier evidence of these factors’ contribution to HTH or because of their potential role as a risk factor for CVD (11). For the above unhealthy lifestyles, we scored 1 for an unhealthy level and 0 for none. Thus, the unhealthy lifestyle score was the total of the points ranging from 0 to 5, with higher scores showing unhealthier lifestyles.

### Assessment of HTH

Single-hypertension is defined as a diastolic/systolic blood pressure ≥ 90/140 mm/Hg or clinically diagnosed with hypertension or taking antihypertensive drugs, but Hcy < 15 μmol/L. Single-HHcy was defined as plasma Hcy ≥ 15 μmol/L with a normal blood pressure. HTH was defined as having a diagnosis of essential hypertension with Hcy ≥ 15 μmol/L.

### Covariates

Demographic characteristics including age, sex, marital status, family income, education and occupation status, and self-reported comorbidities (ischemic heart disease, stroke or diabetes), and laboratory indicators including fasting FPG, TC, TG, LDL-C, HDL-C, and CRP were analyzed in our models as covariates, based on previous research (10).

### Statistical analysis

The mean and standard deviation were used to describe the normal distribution of quantitative variables, and the *t-*test was used to compare the differences. The median and quartile intervals were used to represent the quantitative variables that did not follow the normal distribution, and the *Wilcoxon rank sum* test was used to compare the differences. Categorical variables were expressed as counts and percentages (%), and differences were compared using *Chi-square* test or *Fisher exact probability* method.

Multiple logistic regression models were applied to examine the associations of each lifestyle factor and the overall lifestyle score with health states (including single-HHcy, single-hypertension and HTH) risk. The results were reported as odds ratios (ORs) with 95% CIs. We assigned a median value to each lifestyle score category to test the linear trend. The dose–response relationship between cumulative exposure of behavioral lifestyle and HTH was estimated using a restricted cubic spline (RCS) function. Here, a 1:1 matched case–control study was established using propensity score matching method to further validate the exposure effects of unhealthy lifestyles. The data before and after matching were, respectively, compared to see whether the patients were balanced in important covariates. The difference of the covariates in the two groups before matching was less than 0.05, and the difference of the covariates after matching was greater than 0.06, indicating that the covariates after matching were balanced among groups (see Supplementary Table S1 for details). In this case–control study, to examine the proportion of HTH in the exposed population that theoretically would not have occurred if all participants had adhered to 5 low-risk lifestyle factors (lifestyle score was zero), we calculated the attributable risk proportion (ARP) under the assumption of a causal relationship between lifestyle and HTH risk. The formula for calculating ARP was as follows: $AF=\left(\mathrm{OR}-1\right)/\mathrm{OR}\times 100\%$ .

To verify the robustness of the results, four models were constructed in the sensitivity analysis based on previous researches (10, 21). In model 1, no covariate was adjusted. In model 2, we adjusted for age, sex, education, family income, marital status and occupational status. In model 3, history of prevalent comorbidities (including ischemic heart disease, stroke and diabetes) was further adjusted. In model 4, additionally included the serum FPG, TC, TG, LDL-C and HDL-C and CRP. Meanwhile, we tested the robustness and potential variations in different subgroups stratified by sex (male and female), age groups (<45 years, and ≥45 years) and prevalent comorbidities (yes, and no). Statistical analyses were conducted using STATA version 18.0 (Institute, Gary, NC, United States). A two-sided *p* < 0.05 was considered statistically significant.

## Results

### Population characteristics

Table 1 shows baseline characteristics of participants. A total of 4,012 participants were included, with an average age of 54.6 years (Standard Deviation [SD]: 12.6) and 59.0% female. Among them, 312 (7.8%), 1,072 (26.7%), 1,364 (34.02%), 776 (19.3), 365 (9.1%) and 123 (3.1%) had zero, one, two, three, four and five unhealthy lifestyle factors, respectively. The prevalence rates of single-HHCy, single-hypertension and HTH were 15.6% (627/4012), 15.0% (601/4012) and 19.8% (793/4012), respectively. Those of HTH were more likely to be older, male, to have low family income, less educated, married or cohabitation, farmer, and a higher prevalence of self-reported comorbidities (including ischemic heart disease, stroke and diabetes) than those among healthy group. Additionally, the participants in HTH group have higher levels of serum FPG, TC, TG, LD-C, HDL-C, and CRP. Among the 4,012 participants, 962 (24.0%) were non-current smokers, 1,079 (26.9%) were heavy alcohol drinker, 1974(49.2%) maintained an unhealthy diet, 2,610(65.1) were inactive in exercise and 1,578 (39.3%) had an abnormal BMI. Those unhealthy lifestyles were more prevalent among participants with HTH. Notably, those with HTH were more likely to be exposed to a variety of unhealthy behaviors than healthy population. Supplementary Table S2 provides details about the cumulative exposure of lifestyle factors and includes 32 combinations in total. The most two frequent combinations were the unhealthy diet and inactive exercise group and inactive exercise group, corresponding to 15.7 and 10.2%, respectively.

**Table 1:** Baseline characteristics of participants according to health status (single-HHcy, single-hypertension and HTH)*.

Characteristics	Total	Healthy group	Single-HHcy	Single-hypertension	HTH	*p*-value
*N*	4,012	1991 (49.6)	627 (15.6)	601 (15.0)	793 (19.8)	
Age (years, SD)	54.6 (12.6)	52.7 (12.4)	52.1 (12.6)	56.9 (10.8)	59.6 (12.7)	<0.001
Sex						<0.001
Male	2,368 (59.0)	636 (31.9)	302 (48.2)	262 (43.6)	444 (56.0)	
Female	1,644 (41.0)	1,355 (68.1)	325 (51.8)	339 (56.4)	349 (44.0)	
Family income^†^						0.005
Low	1,559 (38.8)	763 (38.3)	235 (37.5)	217 (36.1)	344 (43.4)	
Medium	1,439 (35.9)	710 (35.7)	237 (37.8)	247 (41.1)	245 (30.9)	
High	1,014 (35.3)	518 (26.0)	155 (24.7)	137 (22.8)	204 (25.7)	
Education						<0.001
Below of high school	2033 (50.7)	1,018 (51.3)	267 (42.6)	287 (47.6)	461 (58.1)	
Ordinary or vocational high school	1,154 (58.7)	543 (27.3)	203 (32.4)	201 (33.4)	207 (26.1)	
Undergraduate or college degree	825 (20.6)	430 (21.6)	157 (25.0)	113 (18.8)	125 (15.8)	
Occupation status						
Wage-laborer	595 (14.8)	318 (16.0)	119 (19.0)	65 (10.8)	93 (11.7)	<0.001
White-collar worker	1,102 (27.5)	606 (30.4)	199 (31.7)	130 (21.6)	167 (21.1)	
Farmer	714 (17.8)	381 (19.1)	71 (11.3)	76 (12.6)	186 (23.5)	
Retiree	1,601 (39.9)	686 (34.5)	238 (38.0)	330 (54.9)	347 (43.8)	
Marital status						
Unmarried	65 (1.6)	24 (1.2)	12 (1.9)	13 (2.2)	16 (2.0)	0.002
Married/cohabitation	3,808 (94.9)	1881 (94.5)	594 (94.7)	571 (95.0)	762 (96.1)	
Divorce/widow	139 (3.5)	86 (4.3)	21 (3.4)	17 (2.8)	15 (1.9)	
Self-reported comorbidities						
Ischemic heart disease	310 (7.7)	117 (5.9)	44 (7.0)	60 (10.0)	89 (11.2)	<0.001
Stroke	114 (2.8)	56 (2.8)	7 (1.1)	17 (2.8)	34 (4.3)	0.005
Diabetes	210 (5.2)	80 (4.0)	19 (3.0)	43 (7.2)	95 (8.6)	<0.001
GLU, mean (SD)	5.7 (1.6)	5.6 (1.7)	5.5 (1.1)	5.6 (1.4)	6.0 (1.5)	<0.001
TC, mean (SD)	4.8 (0.9)	4.7 (0.9)	5.2 (1.0)	4.8 (0.9)	5.0 (1.0)	<0.001
TG, mean (SD)	2.0 (16)	1.7 (1.3)	2.4 (1.7)	1.9 (1.6)	2.5 (2.1)	<0.001
LDL, mean (SD)	2.6 (0.8)	2.6 (0.8)	2.8 (1.0)	2.7 (0.8)	2.6 (0.9)	<0.001
HDL, mean (SD)	1.3 (0.3)	1.4 (0.3)	1.3 (0.3)	1.3 (0.3)	1.2 (0.3)	<0.001
CRP, mean (SD)	5.5 (1.6)	5.5 (1.5)	5.6 (1.4)	5.4 (1.5)	5.7 (2.0)	<0.001
Current smoking	1,079 (26.9)	435 (21.8)	186 (29.7)	140 (23.3)	318 (40.1)	<0.001
Heavy alcohol drinking	962 (24.0)	403 (20.2)	188 (30.0)	135 (22.5)	236 (29.8)	<0.001
Unhealthy diet	1974 (49.2)	864 (43.4)	324 (51.7)	327 (54.4)	459 (57.9)	<0.001
Inactive exercise	2,610 (65.1)	1,260 (63.3)	393 (62.7)	420 (69.9)	537 (67.7)	0.005
BMI ≥24 Kg/m^2^	1,578 (39.3)	632 (31.7)	212 (33.8)	316 (52.6)	418 (52.7)	<0.001
Unhealthy lifestyles score (point)						
0	312 (7.8)	189 (9.5)	48 (7.7)	39 (6.5)	36 (4.6)	<0.001
1	1,072 (26.7)	629 (31.6)	174 (27.7)	119 (19.8)	150 (18.9)	
2	1,364 (34.0)	722 (36.3)	196 (31.3)	219 (36.4)	227 (28.6)	
3	776 (19.3)	307 (15.4)	116 (18.5)	146 (24.3)	207 (26.1)	
4	365 (9.1)	120 (6.0)	76 (12.1)	47 (7.8)	122 (15.4)	
5	123 (3.1)	24 (1.2)	17 (2.7)	31 (5.2)	51 (6.4)	

HHcy, hyper-homocysteine; HTH, H-type hypertension; SD, standard deviation; BMI, body mass index; HTH, H-type hypertension. FPG, fasting blood-glucose; TC, plasma total cholesterol; TG, triglyceride; LDL-C, low density lipoprotein cholesterol; HDL-C, high density lipoprotein cholesterol; CRP, C-reactive protein. *Continuous variables were expressed as mean (95% confidence interval), and categorical variables were expressed as number (percentage). *p*-values were calculated using analysis of variance for continuous variables, and Pearson chi-squared test or Fisher’s exact for categorical variables. ^†^Family income was divided into high, medium, and low level according to triquartile method.

### Associations of unhealthy lifestyle factors with HTH

The associations of single unhealthy lifestyle factors with the risk of three health status were displayed in Supplementary Table S3. The univariate logistic analysis found that, smoking, heavy alcohol drinking, inactive exercise, unhealthy diet and BMI (≥24 Kg/m^2^) were positively associated with HTH. After adjusting for age, sex, marital status, family income, education and occupation status, self-reported comorbidities (including ischemic heart disease, stroke or diabetes), serum FPG, TC, TG LDL-C, HDL-C and CRP, we found the similar positive results, for details, those with HTH had corresponding ORs of 1.29 (for smoking, 95% CI: 1.00–1.66, *p* = 0.046), 1.52 (for unhealthy diet, 95% CI: 1.27–1.82, *p* < 0.001), 1.29 (for inactive exercise, 95% CI: 1.06–1.59, *p* = 0.013), and 2.16 (for BMI, 95% CI: 1.79–2.61, *p* < 0.001), respectively. Additionally, after adjusted for above covariables, only unhealthy diet (OR = 1.49, 95% CI:1.23–1.80) and BMI (OR = 2.38, 95% CI: 1.95–2.90) were positively associated with single-hypertension, while no lifestyles were found associated with Single-HHcy (Supplementary Table S3).

### Effects of cumulative exposure with unhealthy lifestyles on HTH

**Figure 2:**
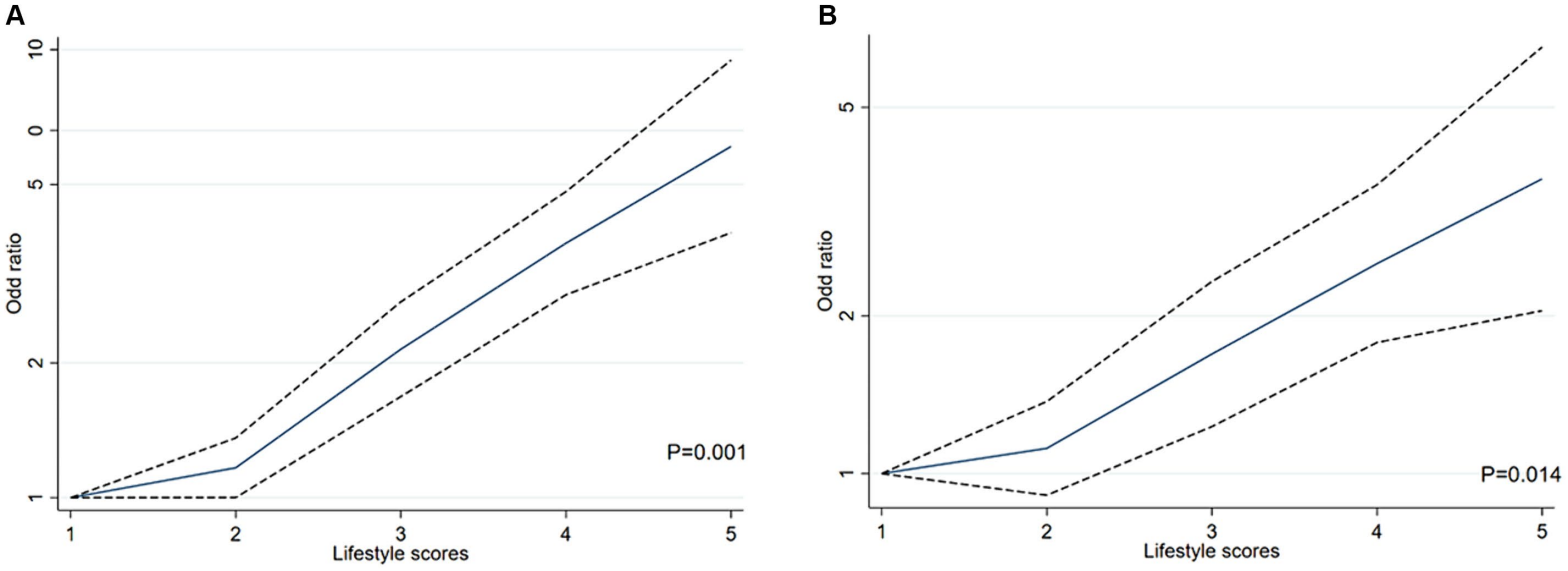
The restricted cubic spline (RCS) for the relationship between unhealthy lifestyle score and H-type hypertension in Chinses adults. The reference value for scores was set as a cut-off value for the first quartile. Three nodes were selected for all models. All models adjusted for age, sex, marital status, family income, education and occupation status, self-reported comorbidities (including ischemic heart disease, stroke or diabetes), serum FPG, TC, TG LDL-C, HDL-C and CRP. **(A)** RCS analysis between unhealthy lifestyle scores and the risk of HTH in cross-sectional study based on 4,012 participants. **(B)** RCS analysis between unhealthy lifestyle scores and the risk of HTH in propensity-score-matched case–control study.

Multiple logistic regression models were carried out to examine the effect of cumulative exposure with unhealthy lifestyles on the risk of HTH (Table 2). In the cross-sectional study based on 4,012 participants, after adjusted for the covariables (including age, sex, marital status, family income, education and occupation status, self-reported comorbidities, serum FPG, TC, TG LDL-C, HDL-C and CRP), when compared with those living favorable lifestyles among healthy group (zero unhealthy lifestyle factors), those with three (OR = 2.12, 95% CI: 1.37–3.27, *p* = 0.001), four (OR = 2.64, 95% CI: 1.61–4.33, *p* < 0.001), or five (OR = 5.08, 95% CI: 2.62–9.84, *p* < 0.001), unhealthy lifestyle factors had higher risk of HTH. Notably, RCS analysis showed that there was a monotonically increasing curve (J-shaped) relationship between unhealthy lifestyle scores and the risk of HTH (*p* = 0.001) (Figure 2A), suggesting that the more the number of unhealthy lifestyle factors, the greater the risk of HTH.

**Table 2:** The effect of cumulative exposure with unhealthy lifestyles on HTH based on 4,012 community population.

Unhealthy lifestyles score (point)	Odd Ratio	95%CI	*p*-value
Model 1			
0	1.00		
1	1.25	0.84–1.86	0.269
2	1.65	1.12–2.43	0.011
3	3.54	2.38–5.27	<0.001
4	5.34	3.45–8.26	<0.001
5	11.16	6.11–20.37	<0.001
Trend for per score point	1.63	1.52–1.75	<0.001
Model 2			
0	1.00		
1	1.11	0.74–1.67	0.611
2	1.26	0.84–1.88	0.259
3	2.52	1.66–3.84	<0.001
4	3.45	2.14–5.58	<0.001
5	8.82	4.65–16.71	<0.001
Trend for per score point	1.50	1.38–1.64	<0.001
Model 3			
0	1.00		
1	1.08	0.72–1.62	0.718
2	1.22	0.81–1.82	0.338
3	2.40	1.57–3.66	<0.001
4	3.27	2.02–5.29	<0.001
5	8.46	4.46–16.04	<0.001
Trend for per score point	1.49	1.37–1.63	<0.001
Model 4			
0	1.00		
1	1.02	0.67–1.55	0.930
2	1.07	0.71–1.62	0.757
3	2.12	1.37–3.27	0.001
4	2.64	1.61–4.33	<0.001
5	5.08	2.62–9.84	<0.001
Trend for per score point	1.40	1.28–1.53	<0.001

In the propensity-score-matched case–control study 1 (614 healthy controls vs. 614 HTH cases), 1,228 participants were included. We confirmed the results of the above cross-sectional study that unhealthy lifestyles had positive cumulative exposure on risk of HTH (Table 3). For details, compared with those living favorable lifestyles, the risk of HTH was increased by 82% (OR = 1.82, 95% CI: 1.03–3.22, *p* = 0.038) and 160% (OR = 2.60, 95% CI: 1.42–4.78, *p* = 0.002) in the groups with three and at least four unhealthy lifestyle factors, respectively. For three unhealthy lifestyle factors group, we found the combination of heavy alcohol consumption, unhealthy diet and BMI ≥24 Kg/m^2^ was most associated with HTH (OR = 7.49, 95%CI:1.12–50.08, *p* = 0.038, Table 4). For four unhealthy lifestyle factors group, the combination of smoking, heavy alcohol consumption, unhealthy diet and BMI ≥24 Kg/m^2^ had the greatest correlation with HTH (OR = 3.75, 95%CI: 1.24–7.38, *p* = 0.015, Table 4). Additionally, trend test analysis prompted that the risk of HTH increased as the number of combined healthy lifestyle factors increased, with participants with four or five unhealthy lifestyle factors having the highest risk of HTH (*P_trend_* < 0.001). We also noticed a significant monotonically increasing (characterized by J-shaped curve) relationship between unhealthy lifestyle scores and the risk of HTH by RCS analysis (*p* = 0.014) (Figure 2B). Table 3 also summarized the ARP for lifestyle score. In case–control study 1, the multivariate ARP of combination of the four/five unhealthy lifestyle factors was 28.23% (95% CI: 6.34–37.86%).

**Table 3:** Cumulative effect of unhealthy lifestyles on HTH based on 1:1 matching case–control studies.

Unhealthy lifestyles score (point)	Healthy group vs. HTH		
	OR	95%CI	*P*-value
0	1.00		
1	1.42	0.81–2.50	0.224
2	1.23	0.71–2.14	0.463
3	1.82	1.03–3.22	0.038
4/5	2.60	1.42–4.78	0.002
Trend for per score point	1.24	1.12–1.37	<0.001
ARP (%, 95CI)	28.23	6.34–37.86	

**Table 4:** Effects of cumulative exposure combination of unhealthy lifestyle on HTH.

Combination of lifestyle factors	OR	95%CI	*p*-value
Score = 3			
Heavy alcohol consumption, unhealthy diet and BMI ≥24 Kg/m^2^	7.49	1.12–50.08	0.038
Unhealthy diet, inactive exercise and BMI ≥24	3.58	1.66–7.69	0.001
Smoking, unhealthy diet and BMI ≥24 Kg/m^2^	3.38	1.22–9.41	0.020
Smoking, heavy alcohol consumption and unhealthy diet	1.71	0.11–26.41	0.702
Smoking, heavy alcohol consumption and inactive exercise	0.23	0.03–1.98	0.196
Smoking, heavy alcohol consumption and BMI ≥24 Kg/m^2^	1.00	1.00–1.00	1.000
Smoking, unhealthy diet and inactive exercise	1.48	0.71–3.07	0.292
Smoking, inactive exercise and BMI ≥24 Kg/m^2^	3.11	0.89–14.63	0.651
Heavy alcohol consumption, unhealthy diet and inactive exercise	1.48	0.59–3.72	0.404
Heavy alcohol consumption, inactive exercise and BMI ≥24 Kg/m^2^	1.00	1.00–1.00	1.000
Score = 4			
Smoking, heavy alcohol consumption, unhealthy diet and BMI ≥24 Kg/m^2^	3.75	1.24–7.38	0.015
Smoking, heavy alcohol consumption, inactive exercise and BMI ≥24 Kg/m^2^	3.26	1.01–10.51	0.048
Heavy alcohol consumption, unhealthy diet, inactive exercise and BMI ≥24 Kg/m^2^	3.17	1.13–8.89	0.028
Smoking, unhealthy diet, inactive exercise and BMI ≥24 Kg/m^2^	3.03	0.79–17.90	0.097
Smoking, heavy alcohol consumption, unhealthy diet and inactive exercise	1.18	0.54–2.57	0.670
Score = 5			
Smoking, heavy alcohol consumption, unhealthy diet, inactive exercise and BMI ≥24 Kg/m^2^	3.41	1.50–7.80	0.004

### Sensitivity and subgroup analysis

In sensitivity analyses, we constructed several models, and found the results remained similar in all sensitivity analyses (Table 2). Then, we conducted subgroup analysis. Supplementary Table S4 shows the results stratified by sex, age group, and self-reported comorbidity groups, which were not materially changed with those of the main analyses. For instance, compared with those of zero unhealthy lifestyle factor, the cumulative exposure effect of unhealthy lifestyle (five unhealthy lifestyle factors) on risk of HTH were stronger in males (OR = 4.69, 95%CI: 1.60–8.38) than in females, and in older (OR = 4.86, 95%CI: 2.30–10.30) than younger adults. Cumulative exposure effect was more pronounced in those with comorbidities than those without, with an adjusted OR of 2.52 (95% CI: 1.41–4.52 *p* = 0.002) obtained for three unhealthy lifestyle factors, 3.57 (95% CI: 1.89–6.73, *p* < 0.001) for four unhealthy lifestyle factors and 6.81 (95% CI: 3.19–14.57, *p* < 0.001) for five unhealthy lifestyle factors, respectively (Supplementary Table S4).

## Discussion

In this study, we found that an unhealthy lifestyle score defined by five high-risk lifestyle factors (including smoking, heavy alcohol drinking, unhealthy diet, inactive exercise and high BMI) was significantly associated with a higher risk of HTH, suggesting that there was a positive cumulative exposure of unhealthy lifestyle on HTH among Chinese adults, with the largest effect combination being heavy alcohol consumption, unhealthy diet and BMI ≥24 Kg/m^2^. Participants who had three or more unhealthy lifestyle factors exhibited an increase in their risk of HTH, ranging from 82 to 408%, compared to those having no unhealthy lifestyles (0 unhealthy lifestyle factor). These unhealthy lifestyle factors explained about 28.23% of the HTH risk.

In our study, we found that smokers had a higher risk of developing H-hypertension than non-smokers. Epidemiological studies have shown that smokers tend to have lower levels of the B-vitamins, such as folate, vitamin B6 and vitamin B12, which affect homocysteine levels by controlling co-factors or co-substrates of homocysteine metabolizing enzymes (22, 23). And, smoking has been shown to increase the risk of vascular damage by increasing sympathetic tone, platelet viscosity and reactivity, free radical production, endothelial damage, or elevated arterial pressure (24). Data from eight prospective cohort studies involving 70,130 participants and 21,238 cases of hypertension, suggested that smoking cessation did not increase the risk of hypertension (25). Therefore, there is an urgent need to provide effective strategies to encourage smokers to quit, especially those with H-type hypertension, and thus reduce their risk of developing CVD. Additionally, alcohol consumption is an important preventable and modifiable cause of non-communicable disease, and increased the risk of hypertension in a positive dose-dependent manner (26). In present study, univariate analysis showed that heavy alcohol consumption level (average daily alcohol drinks ≥2) was associated with increased risk of HTH (OR = 1.67, 95% CI: 1.38–2.01). However, after adjustment for potential confounders (sex and age, etc.), no significant increase in HTH prevalence risk was observed (OR = 1.06, 95% CI: 0.85–1.33). Gender is an important modifier of the alcohol threshold level for harm. The Physicians’ Health study reported a definite protective effect on hypertension in women who drank 2 ~ 4 drinks per week up to one drink per day, but the relationship was linear in men (27). In our study, the proportion of male population in HTH group (56.0%) was significantly lower than that in control group (31.9%). This sex difference May be due to differences in testosterone regulation, which results in higher renal cysteine beta-synthetase (CBS) activity in men than in women, and CBS catalyzes an important pathway for intracellular homocysteine metabolism (28). Thus, as there is no safe amount of alcohol to drink, it is best to aim for abstinence in the first instance, especially for males with HTH.

Our present study found that the unhealthy diet did not differ statistically between the HHcy group and the normal Hcy group after adjusted for other potential confounders, which was contrary to some studies showing that higher fruit and vegetable intake was associated with lower Hcy levels (29, 30). However, other studies have shown that vegetarians were at much higher risk of HHcy than non-vegetarians (31, 32). As is well-known, the intake of a variety of foods is more in line with human physiological characteristics. In addition, foods rich in B vitamins are not only vegetables and fruits, but also unprocessed meats, animal livers, and fish (33). Therefore, appropriately increasing the intake of vegetables and fruits, as part of a balanced diet, can benefit healthy people, while B vitamin supplementation may have a more direct effect on HHcy patients. In addition, greater daily physical activity is associated with lower homocysteine levels and that active exercise programs could positively control homocysteine level (34). However, our study found no relationship of inactive exercise with HHcy, which was similar with another study (35). These conflicting conclusions May be due to differences and flaws in study design, which require further validation in prospective studies. In fact, exercise training prevents the development of atherosclerosis through SIRT1 activation and oxidative stress inhibition under HHcy situation (36). In this study, we found inactive exercise increased the risk of HTH, further validating the results of previous study from another region in China (37). Thus, in the prevention and treatment of HTH and CVD, active physical exercise, especially aerobic exercise, should be put on the agenda. Besides, high BMI has been associated with HTH, and being obese or overweight (BMI ≥24 Kg/m^2^) was a potential risk factor for HTH (38). Our research supports this view.

Previous observational studies have demonstrated that individual lifestyle factors are of critical importance in the progression of HTH (11, 39). However, it is worth noting that from the perspective of life course theory (12), there is a cumulative effect of risk factors in the pathogenesis of chronic diseases. For instance, a systematic review and meta-analysis of 142 prospective cohort showed that those with the healthiest lifestyles had lower risks of morbidity and mortality of CVD, compared with the participants with the least-healthy lifestyles (including cigarette smoking, alcohol consumption, physical activity, diet, and BMI) (14). Another study from Netherlands Cohort Study found that a significant healthy lifestyle score (smoking, BMI, physical activity, Mediterranean diet adherence, and alcohol intake) was significantly inversely associated with risk of esophageal and gastric cancer, in a linear fashion (13). A 12.2-year follow-up study of UK Biobank indicated that compared with the very unhealthy group (smoking, alcohol consumption, diet, and physical activity), the very healthy group had a 41% reduction in the risk for cardiometabolic multimorbidity in hypertensive patients and a 32–50% reduction in the risk for specific cardiometabolic disease (40). Therefore, it is of great importance to consider multiple healthy lifestyle factors when investigating the relationship of lifestyles with HTH because different behaviors tend to promote each other. For instance, most people who drink also smoke (41). To the best of our knowledge, however, little research has looked the relationship between cumulative exposures of lifestyle factors and HTH. Notably, our study provides important and new information on this issue, in which having a combination of unhealthy lifestyles (involving smoking, heavy alcohol consumption, inactive exercise, unhealthy diet and abnormal BMI) May higher the risk of HTH among the general population in China. Critically, these unhealthy lifestyle factors explained about 28.23% for the HTH risk, suggesting that avoiding these unhealthy lifestyle factors can reduce the risk of HTH by28.23%. Thus, lifestyle modifications, such as smoking cessation, reducing heavy alcohol drinking, regular physical activity, complement the nutritional approach and keeping a normal BMI to enhance Hcy metabolism (42), has great potential in the primary prevention of HTH.

In this study, we included baseline sex (males vs. female), age (<45 years vs. ≥45 years) and self-reported comorbidity groups (yes vs. no) in a subgroup analysis, which were not materially changed with those of the main analyses. For instance, cumulative exposure effect if unhealthy lifestyles were more pronounced in females than males, in elders than youngers and in those with comorbidities than those without. Gender roles and social norms lead to different lifestyle risk factors for males and females, for example, males are more likely to have unhealthy lifestyles like cigarettes smoking, heavy alcohol consumption, eating poorly and not exercising (43). And, Hcy levels increased with age, and total HHcy prevalence has been reported to be higher in the elderly than in youngers, due to nutritional causes or metabolic changes common to old age and poor nutritional absorption (44). In general, patients under comorbidity status have insufficient awareness of the risk of unhealthy lifestyles (45). Thus, these findings suggest that we should also take unhealthy behaviors change into account, especially in the males, elder and those with comorbidities, when preventing and screening for the HTH, thereby preventing the development of cardiovascular disease.

The study we conducted has a number of strengths. To the best of our knowledge, our study is the first to explore the effect of cumulative exposure with unhealthy lifestyles on HTH using a combined unhealthy lifestyle enabled us to comprehensively characterize an individual’s profile, and confirmed that the combination of heavy alcohol consumption, unhealthy diet and BMI ≥24 Kg/m^2^ was the most associated with HTH. Furthermore, we use a PSM case–control study to verify the positive association between lifestyle score and HTH, which can ensure the objectivity of the study, using similar covariate distributions to construct treatment and control groups without affecting study outcomes (15). As a semi-parametric method, PSM has fewer formal restrictions on the processing of model functions and fewer distribution assumptions for error terms, which increases the possibility of reasonable matching between the treatment group and the control group. Compared with the traditional method, when dealing with the problems such as multiple confounding factors or stratification, the matching has the possibility, and the calculation amount is greatly reduced, which provides an efficient and appropriate matching for the research (15). However, there were still some potential limitations that should be considered in interpretation of results. Firstly, many variables were self-reported and only evaluated at a point in time, so there May be recall or evaluation biases. For example, we used a composite index that included a variety of foods to assess unhealthy diet, however, it was not possible to completely avoid recall bias. Thus, the generalisability of our findings should be interpreted with caution. Besides, the lifestyle was only assessed by questionnaire at baseline. Future studies with repeated and objective assessment, such as wearables to assess individual exercise metabolism (46), will be necessary. Additionally, although lifestyle score was evaluated as a composite index (including smoking, heavy alcohol drinking, unhealthy diet, inactive exercise, and high BMI) in our study, other possible lifestyles (e.g., sitting/sedentary behaviors) associated with risk of HTH were not considered. Second, participants in our study were from Hunan province, China, although the sample of this special population is considered large, the generalizability of our findings May be constrained in other regions of China. Thus, future multi-province or multinational monitoring studies are necessary to confirm our findings. Additionally, due to the case–control design of this study, causality cannot be inferred. Therefore, larger prospective investigations are required in the future to confirm the current findings. Finally, in present study, we focused on the association of unhealthy lifestyles with H-type hypertension, rather than with other clinical phenotypes such as ventricular hypertrophy in hypertension, which is a direct effect on the heart. In the next studies, we will consider using echocardiography to evaluate ventricular hypertrophy and explore the influence of unhealthy lifestyles on its occurrence.

In conclusion, our findings add new evidence to this field that cumulative exposure of unhealthy lifestyles (including smoking, heavy alcohol drinking, unhealthy diet, inactive exercise and high BMI) is consistently associated with higher risk of HTH among Chinses adults, with the largest effect combination being heavy alcohol consumption, unhealthy diet and BMI ≥24 Kg/m^2^. These findings highlight that the effectiveness of comprehensive lifestyle modification in the prevention of HTH could be adopted to and reduce the risk of developing long-term CVD. Further long-term longitudinal studies are needed to evaluate the potential benefits of other lifestyles within this population to reduce the burden of disease.

## Data Availability

The original contributions presented in the study are included in the article/Supplementary material, further inquiries can be directed to the corresponding author.

## References

[1] Mensah GA Roth GA Fuster V. The global burden of cardiovascular diseases and risk factors: 2020 and beyond. J Am Coll Cardiol. (2019) 74:2529–32. doi: 10.1016/j.jacc.2019.10.009 31727292

[2] Vaduganathan M Mensah GA Turco JV Fuster V Roth GA. The global burden of cardiovascular diseases and risk: a compass for future health. J Am Coll Cardiol. (2022) 80:2361–71. doi: 10.1016/j.jacc.2022.11.005 36368511

[3] Wu DF Yin RX Deng JL. Homocysteine, hyperhomocysteinemia and H-type hypertension. Eur J Prev Cardiol. (2024) 31:1092–103. doi: 10.1093/eurjpc/zwae022, PMID: 38236144

[4] Li JP Huo Y Liu P. Efficacy and safety of Enalapril-folate acid tablets in lowering blood pressure and plasma homocysteine. Beijing Da Xue Xue Bao. (2007) 39:614–8. doi: 10.3321/j.issn:1671-167x.2007.06.015 PMID: 18087553

[5] Hu DY Xu XP. Prevention of stroke relies on valid control "H" type hypertension. Zhonghua Nei Ke Za Zhi. (2008) 47:976–7. doi: 10.3321/j.issn:0578-1426.2008.12.005 PMID: 19134296

[6] Zhang ZY Gao G Li Y Si SC Wang JY Wei ZY. Research progress on the correlation between hypertension combined with high homocysteine and cardio-cerebrovascular disease. Chin J Hypertens. (2021) 29:622–8. doi: 10.16439/j.issn.1673-7245.2021.07.006

[7] Li T Liu X Diao S Kong Y Duan X Yang S. H-type hypertension is a risk factor for cerebral small-vessel disease. Biomed Res Int. (2020) 2020:6498903. doi: 10.1155/2020/6498903 32090105 PMC7029257

[8] Stea TH Mansoor MA Wandel M Uglem S Frølich W. Changes in predictors and status of homocysteine in young male adults after a dietary intervention with vegetables, fruits and bread. Eur J Nutr. (2008) 47:201–9. doi: 10.1007/s00394-008-0714-y, PMID: 18521531

[9] Liu XD Gao B Sun D Shi M Ma YY Liu ZR. Prevalence of hyperhomocysteinaemia and some of its major determinants in Shaanxi Province, China: a cross-sectional study. Br J Nutr. (2015) 113:691–8. doi: 10.1017/S0007114514004218, PMID: 25634595

[10] Yang Y Zeng Y Yuan S Xie M Dong Y Li J. Prevalence and risk factors for hyperhomocysteinemia: a population-based cross-sectional study from Hunan. China BMJ Open. (2021) 11:e048575. doi: 10.1136/bmjopen-2020-048575, PMID: 34872994 PMC8650492

[11] Zhang C Li J Zhou J Zheng Q Dong R Xing E. Effect of MTHFRC677 T gene polymorphism on early morning blood pressure in elderly female patients with H-type hypertension. Contrast Media Mol Imaging. (2022) 2022:2530388. doi: 10.1155/2022/2530388, PMID: 36299829 PMC9576431

[12] Lynch J Smith GD. A life course approach to chronic disease epidemiology. Annu Rev Public Health. (2005) 26:1–35. doi: 10.1146/annurev.publhealth.26.021304.144505 15760279

[13] van den Brandt PA. The impact of a healthy lifestyle on the risk of esophageal and gastric cancer subtypes. Eur J Epidemiol. (2022) 37:931–45. doi: 10.1007/s10654-022-00899-w, PMID: 35982188 PMC9529711

[14] Zhang YB Pan XF Chen J Cao A Xia L Zhang Y. Combined lifestyle factors, all-cause mortality and cardiovascular disease: a systematic review and meta-analysis of prospective cohort studies. J Epidemiol Community Health. (2021) 75:92–9. doi: 10.1136/jech-2020-214050, PMID: 32892156

[15] Borah BJ Moriarty JP Crown WH Doshi JA. Applications of propensity score methods in observational comparative effectiveness and safety research: where have we come and where should we go? J Comp Eff Res. (2014) 3:63–78. doi: 10.2217/cer.13.89, PMID: 24266593

[16] Cohen E Margalit I Shochat T Goldberg E Krause I. Gender differences in homocysteine concentrations, a population-based cross-sectional study. Nutr Metab Cardiovasc Dis. (2019) 29:9–14. doi: 10.1016/j.numecd.2018.09.003, PMID: 30459075

[17] Zeng Y Li FF Yuan SQ Tang HK Zhou JH He QY. Prevalence of Hyperhomocysteinemia in China: an updated meta-analysis. Biology. (2021) 10:959. doi: 10.3390/biology10100959 34681058 PMC8533293

[18] Li M Hu L Zhou W Wang T Zhu L Zhai Z. Non-linear association between blood lead and hyperhomocysteinemia among adults in the United States. Sci Rep. (2020) 10:17166. doi: 10.1038/s41598-020-74268-6, PMID: 33051568 PMC7553908

[19] Wang Z Zou Z Yang Z Dong Y Ma J. Association between exposure to the Chinese famine during infancy and the risk of self-reported chronic lung diseases in adulthood: a cross-sectional study. BMJ Open. (2017) 7:e015476. doi: 10.1136/bmjopen-2016-015476, PMID: 28576899 PMC5623412

[20] Craig R. Mindell J. Hirani V., Health survey for England 2008. The Health and Social Care Information Centre: physical activity and fitness. (2009).

[21] Hu Z Hou QZ Zhao S Liang Y Shen A. Structural and functional changes of the carotid artery and their relationship with subclinical inflammation in patients with H-type hypertension. Nan Fang Yi Ke Da Xue Xue Bao. (2012) 32:1175–8. doi: 10.3969/j.issn.1673-4254.2012.08.23 PMID: 22931616

[22] O'Callaghan P Meleady R Fitzgerald T Graham I. Smoking and plasma homocysteine. Eur Heart J. (2002) 23:1580–6. doi: 10.1053/euhj.2002.3172 12323157

[23] McCarty MF. Increased homocyst(e)ine associated with smoking, chronic inflammation, and aging may reflect acute-phase induction of pyridoxal phosphatase activity. Med Hypotheses. (2000) 55:289–93. doi: 10.1054/mehy.1999.1032, PMID: 11000053

[24] Sleight P. Smoking and hypertension. Clin Exp Hypertens. (1993) 15:1181–92. doi: 10.3109/10641969309037104 8268884

[25] Sun AL Li GJ Wei T. Effects of smoking cessation on the risk of hypertension: a meta-analysis. Zhonghua Yi Xue Za Zhi. (2019) 99:2068–72. doi: 10.3760/cma.j.issn.0376-2491.2019.26.013, PMID: 31315379

[26] Taylor B Irving HM Baliunas D Roerecke M Patra J Mohapatra S. Alcohol and hypertension: gender differences in dose-response relationships determined through systematic review and meta-analysis. Addiction. (2009) 104:1981–90. doi: 10.1111/j.1360-0443.2009.02694.x, PMID: 19804464

[27] Sesso HD Cook NR Buring JE Manson JE Gaziano JM. Alcohol consumption and the risk of hypertension in women and men. Hypertension. (2008) 51:1080–7. doi: 10.1161/HYPERTENSIONAHA.107.104968 18259032

[28] Vitvitsky V Prudova A Stabler S Dayal S Lentz SR Banerjee R. Testosterone regulation of renal cystathionine beta-synthase: implications for sex-dependent differences in plasma homocysteine levels. Am J Physiol Renal Physiol. (2007) 293:F594–600. doi: 10.1152/ajprenal.00171.2007, PMID: 17537983

[29] Gao X Yao M McCrory MA Ma G Li Y Roberts SB. Dietary pattern is associated with homocysteine and B vitamin status in an urban Chinese population. J Nutr. (2003) 133:3636–42. doi: 10.1093/jn/133.11.3636, PMID: 14608087

[30] Verly E Jr Steluti J Fisberg RM Marchioni DM. A quantile regression approach can reveal the effect of fruit and vegetable consumption on plasma homocysteine levels. PLoS One. (2014) 9:e111619. doi: 10.1371/journal.pone.0111619, PMID: 25365261 PMC4218785

[31] Bissoli L Di Francesco V Ballarin A Mandragona R Trespidi R Brocco G. Effect of vegetarian diet on homocysteine levels. Ann Nutr Metab. (2002) 46:73–9. doi: 10.1159/000057644 12011576

[32] Krajcovicová-Kudlácková M Blazícek P. Nutritional determinants of homocysteinemia. Cas Lek Cesk. (2002) 141:417–20. Available at: https://pubmed.ncbi.nlm.nih.gov/12238029/12238029

[33] Konstantinova SV Vollset SE Berstad P Ueland PM Drevon CA Refsum H. Dietary predictors of plasma total homocysteine in the Hordaland homocysteine study. Br J Nutr. (2007) 98:201–10. doi: 10.1017/S0007114507691788, PMID: 17391553

[34] Silva A d S e da Mota MPG. Effects of physical activity and training programs on plasma homocysteine levels: a systematic review. Amino Acids. (2014) 46:1795–804. doi: 10.1007/s00726-014-1741-z 24770903

[35] Boreham CA Kennedy RA Murphy MH Tully M Wallace WF Young I. Training effects of short bouts of stair climbing on cardiorespiratory fitness, blood lipids, and homocysteine in sedentary young women. Br J Sports Med. (2005) 39:590–3. doi: 10.1136/bjsm.2002.001131, PMID: 16118293 PMC1725304

[36] Chan SH Hung CH Shih JY Chu PM Cheng YH Lin HC. Exercise intervention attenuates hyperhomocysteinemia-induced aortic endothelial oxidative injury by regulating SIRT1 through mitigating NADPH oxidase/LOX-1 signaling. Redox Biol. (2018) 14:116–25. doi: 10.1016/j.redox.2017.08.016, PMID: 28888894 PMC5596261

[37] Wang W Ji P Wang Y Guo H Bian R Xu J. Prevalence of hyperhomocysteinemia and its associated factors in patients with primary hypertension in Chinese urban communities: a cross-sectional study from Nanjing. Clin Exp Hypertens. (2018) 40:495–500. doi: 10.1080/10641963.2017.1403621, PMID: 29172835

[38] Wang J Du J Fan R. Exploration of the risk factors of essential hypertension with hyperhomocysteinemia: a hospital-based study and nomogram analysis. Clinics. (2021) 76:e2233. doi: 10.6061/clinics/2021/e2233, PMID: 33503187 PMC7798116

[39] Du S Hong X Yang Y Ding Z Yu T. Association between body fat percentage and H-type hypertension in postmenopausal women. Front Public Health. (2022) 10:950805. doi: 10.3389/fpubh.2022.950805, PMID: 35937205 PMC9354540

[40] Xie H Li J Zhu X Li J Yin J Ma T. Association between healthy lifestyle and the occurrence of cardiometabolic multimorbidity in hypertensive patients: a prospective cohort study of UK biobank. Cardiovasc Diabetol. (2022) 21:199. doi: 10.1186/s12933-022-01632-3, PMID: 36183084 PMC9526960

[41] Johnson PB Boles SM Vaughan R Kleber HD. The co-occurrence of smoking and binge drinking in adolescence. Addict Behav. (2000) 25:779–83. doi: 10.1016/S0306-4603(99)00066-0, PMID: 11023019

[42] González-Lamuño D Arrieta-Blanco FJ Fuentes ED Forga-Visa MT Morales-Conejo M Peña-Quintana L. Hyperhomocysteinemia in adult patients: a treatable metabolic condition. Nutrients. (2023) 16:135. doi: 10.3390/nu16010135, PMID: 38201964 PMC10780827

[43] Li L He J Ouyang F Qiu D Li Y Luo D. Sociodemographic disparity in health-related behaviours and dietary habits among public workers in China: a cross-sectional study. BMJ Open. (2021) 11:e047462. doi: 10.1136/bmjopen-2020-047462, PMID: 34344677 PMC8336184

[44] Janson JJ Galarza CR Murúa A Quintana I Przygoda PA Waisman G. Prevalence of hyperhomocysteinemia in an elderly population. Am J Hypertens. (2002) 15:394–7. doi: 10.1016/S0895-7061(01)02165-3, PMID: 12022240

[45] Wu Z Li ZR Dai YQ Zhu FY Tan JX Wan LH. Relationship between risk perception and lifestyle in ischemic stroke patients with H-type hypertension. Ann Palliat Med. (2020) 9:3731–41. doi: 10.21037/apm-20-2012, PMID: 33302645

[46] Stamatakis E Ahmadi MN Gill JMR Thøgersen-Ntoumani C Gibala MJ Doherty A. Association of wearable device-measured vigorous intermittent lifestyle physical activity with mortality. Nat Med. (2022) 28:2521–9. doi: 10.1038/s41591-022-02100-x, PMID: 36482104 PMC9800274

