# Supplementary material for: The effect of cumulative exposure with unhealthy lifestyles on the H-type hypertension among Chinese adults: a community-based, propensity-score-matched, and case–control study
Source: Front Nutr. 2024 Sep 18;11:1470788. doi: 10.3389/fnut.2024.1470788 (PMC11445168; doi:10.3389/fnut.2024.1470788)
Supplement: Supplementary file 1 [file Data_Sheet_1.docx]

**Supplementary methods of assessment of unhealthy diet.**

We evaluated dietary status using a more recent dietary recommendation for blood pressure and combining with traditional Chinese eating habits, which considered adequate consumption of fresh fruit, fresh vegetables, unprocessed meats (including red meat, fish or shellfish), reduced consumption of high-fat, high-salt and sugar-sweetened food. The unhealthy diet score was dichotomized as 1 = fewer than 4 of the healthy food items, and 0 = at least 4 of the healthy food items.

**Table 1 for supplementary method**. Components of more recent dietary recommendations for blood pressure health.

| **Diet complement** | **Intake goal** |
| --- | --- |
| Fresh vegetable | Consumption every day |
| Fresh fruit | Consumption every day |
| Unprocessed meats | More than two times/week |
| High-fat diet | ≤ Once a week |
| High-salt diet | ≤ Once a week |
| Sugar-sweetened food | No consumption |

**Table S1**. Distribution of characteristics among HTH and healthy group.

|  |  | **Overall data** | | | | **Propensity 1:1 Matching** | | |
| --- | --- | --- | --- | --- | --- | --- | --- | --- |
| **Variables** |  | **Control**  **(n=1991)** | | **Case**  **(n=793)** | ***p-value*** | **Control**  **(n=614)** | **Case**  **(n=614)** | ***p-value*** |
| Region |  |  | |  | 0.599 |  |  | 0.436 |
| Changsha |  | 969(48.7) | | 410(51.7) |  | 305(49.7) | 317(51.6) |  |
| Zhuzhou |  | 409(20.5) | | 147(18.5) |  | 130(21.2) | 123(20.0) |  |
| Hengyang |  | 256(12.7) | | 108(13.6) |  | 94(15.3) | 83(13.5) |  |
| Yueyang |  | 233(11.7) | | 86(10.8) |  | 61(9.9) | 53(8.6) |  |
| Yongzhou |  | 72(3.6) | | 25(3.2) |  | 13(2.1) | 21(3.4) |  |
| Xiangxi |  | 52(2.6) | | 17(2.1) |  | 11(1.8) | 17(2.8) |  |
| Age (years, SD) |  | 52.7(12.4) | | 59.6(12.7) | <0.001 | 57.5 (11.6) | 58.4(12.8) | 0.267 |
| Sex |  |  | |  | <0.001 |  |  | 0.648 |
| Male |  | 636(31.9) | | 444(56.0) |  | 307 (50.0) | 299 (48.7) |  |
| Female |  | 1355(68.1) | | 349(44.0) |  | 307 (50.0) | 315 (51.3) |  |
| Family income |  |  | |  | 0.111 |  |  | 0.216 |
| Low |  | 763 (38.3) | | 344(43.4) |  | 262 (42.7) | 263 (42.8) |  |
| Medium |  | 710 (35.7) | | 245(30.9) |  | 230 (37.5) | 194 (31.6) |  |
| High |  | 518 (26.0) | | 204(25.7) |  | 122 (19.9) | 157 (25.6) |  |
| Education |  |  | |  | <0.001 |  |  | 0.290 |
| Below of High School | | | 1018(51.1) | 461(58.1) |  | 391 (63.7) | 353(57.5) |  |
| Ordinary/Vocational high school |  | 543 (27.3) | | 207(26.1) |  | 139 (22.6) | 159(25.9) |  |
| Undergraduate/college degree |  | 430 (21.6) | | 125(15.8) |  | 84 (13.7) | 102(16.6) |  |
| Marital status |  |  | |  | <0.001 |  |  | 1.000 |
| Unmarried |  | 24 (1.2) | | 16 (2.0) |  | 5 (0.8) | 8 (1.3) |  |
| Married/Cohabitation |  | 1881 (94.5) | | 762(96.1) |  | 597 (97.2) | 594(96.7) |  |
| Divorce/Widow |  | 86(4.3) | | 15(1.9) |  | 12(2.0) | 12(2.0) |  |
| Occupation status |  |  | |  | <0.001 |  |  | 0.826 |
| Wage-labourer |  | 318 (16.0) | | 93 (11.7) |  | 68 (11.1) | 72 (11.7) |  |
| White-collar worker |  | 606 (30.4) | | 167 (21.1) |  | 132 (21.5) | 133(21.7) |  |
| Farmer |  | 381 (19.1) | | 186 (23.5) |  | 173 (28.2) | 151(24.6) |  |
| Retiree |  | 686 (34.5) | | 347(43.8) |  | 241 (39.3) | 258(42.0) |  |
| Self-reported comorbidities | | | |  |  |  |  |  |
| Ischemic heart disease | | | 117(5.9) | 89(11.2) | <0.001 | 44(7.2) | 62(10.1) | 0.067 |
| Stroke |  | 56(2.8) | | 34(4.3) | <0.001 | 21(3.4) | 22(3.6) | 0.877 |
| Diabetes |  | 80(4.0) | | 95(8.6) | <0.001 | 31(5.1) | 50(8.1) | 0.091 |
| FPG |  | 5.6 (1.7) | | 6.0 (1.5) | <0.001 | 5.7 (1.8) | 5.9 (1.4) | 0.056 |
| TG |  | 4.7 (0.9) | | 5.0 (1.0) | <0.001 | 4.8 (0.9) | 4.8 (0.9) | 0.133 |
| TC |  | 1.7 (1.3) | | 2.5 (2.1) | <0.001 | 1.9 (1.3) | 2.1 (1.36) | 0.970 |
| LDL-C |  | 2.6(0.8) | | 2.6 (0.9) | <0.001 | 2.6 (0.8) | 2.6 (0.8) | 0.454 |
| HDL-C |  | 1.4 (0.3) | | 1.2 (0.3) | <0.001 | 1.3 (0.3) | 1.3 (0.3) | 0.390 |
| CRP |  | 5.5 (1.5) | | 5.7 (2.0) | <0.001 | 5.5 (1.5) | 5.5 (1.8) | 0.931 |

HTH = H-type hypertension; SD= standard deviation; FPG = fasting blood-glucose, TC = plasma total cholesterol, TG = triglyceride, LDL-C = low density lipoprotein cholesterol, HDL-C = high density lipoprotein cholesterol, CRP = C-reactive protein.

**Table S2.** Details about combination of lifestyle factors.

| **Combination of lifestyle factors** | **Frequency** | **Proportion (%)** |
| --- | --- | --- |
| Unhealthy diet and inactive exercise | 629 | 15.68 |
| Inactive exercise | 411 | 10.24 |
| Unhealthy diet, inactive exercise and inactive exercise | 398 | 9.92 |
| Unhealthy diet | 274 | 6.83 |
| None | 223 | 5.56 |
| Inactive exercise and BMI ≥24 Kg/m^2^ | 215 | 5.36 |
| Unhealthy diet and BMI ≥24 Kg/m^2^ | 202 | 5.03 |
| Smoking, heavy alcohol consumption, unhealthy diet, inactive exercise and BMI ≥24 Kg/m^2^ | 156 | 3.89 |
| Smoking, unhealthy diet and inactive exercise | 145 | 3.61 |
| Smoking, heavy alcohol consumption, unhealthy diet and inactive exercise | 115 | 2.87 |
| Smoking, unhealthy diet, inactive exercise and BMI ≥24 Kg/m^2^ | 114 | 2.84 |
| Smoking, heavy alcohol consumption and unhealthy diet | 107 | 2.67 |
| BMI ≥24 Kg/m^2^ | 103 | 2.57 |
| Heavy alcohol consumption and Unhealthy diet | 77 | 1.92 |
| Smoking, unhealthy diet and BMI ≥24 Kg/m^2^ | 69 | 1.72 |
| Smoking and Unhealthy diet | 68 | 1.69 |
| Heavy alcohol consumption, unhealthy diet and inactive exercise | 68 | 1.69 |
| Smoking, heavy alcohol consumption, unhealthy diet and BMI ≥24 Kg/m^2^ | 66 | 1.65 |
| Smoking and inactive exercise | 62 | 1.55 |
| Heavy alcohol consumption and inactive exercise | 61 | 1.52 |
| Heavy alcohol consumption, unhealthy diet, inactive exercise and BMI ≥24 Kg/m^2^ | 60 | 1.50 |
| Heavy alcohol cunsumption | 48 | 1.20 |
| Heavy alcohol consumption, unhealthy diet and BMI ≥24 Kg/m^2^ | 44 | 1.10 |
| Smoking, heavy alcohol consumption, inactive exercise and BMI ≥24 Kg/m^2^ | 43 | 1.07 |
| Smoking | 35 | 0.87 |
| Heavy alcohol consumption and BMI ≥24 Kg/m^2^ | 31 | 0.77 |
| Smoking and Heavy alcohol cunsumption | 26 | 0.65 |
| Heavy alcohol consumption, inactive exercise and BMI ≥24 Kg/m^2^ | 26 | 0.65 |
| Smoking, heavy alcohol consumption and inactive exercise | 22 | 0.55 |
| Smoking, inactive exercise and BMI ≥24 Kg/m^2^ | 22 | 0.55 |
| Smoking and BMI ≥24 Kg/m^2^ | 17 | 0.42 |
| Smoking, heavy alcohol consumption and BMI ≥24 Kg/m^2^ | 12 | 0.30 |

**Table S3**. Associations of unhealthy lifestyle factors with single-HHcy, single Hypertension or HTH.

|  |  | | **OR, 95%CI** | | |
| --- | --- | --- | --- | --- | --- |
| **Models*** |  | **Single-HHcy** | | **Single-hypertension** | **HTH** |
| **Model 1** |  |  | |  |  |
| Smoking |  | 1.51,1.23-1.85 | | 1.09,0.87-1.35 | 2.39,2.01-2.86 |
| Heavy alcohol drinking |  | 1.69,1.38-2.07 | | 1.14,0.92-1.42 | 1.67,1.38-2.01 |
| Unhealthy diet |  | 1.39,1.17-1.37 | | 1.56,1.30-1.87 | 1.79,1.52-2.12 |
| Inactive exercise |  | 0.97,0.81-1.17 | | 1.34,1.11-1.64 | 1.22,1.02-1.45 |
| BMI≥24Kg/m^2^ |  | 1.10,0.91-1.33 | | 2.38,1.98-2.87 | 2.40,2.03-2.84 |
| **Model 2** |  |  | |  |  |
| Smoking |  | 0.90,0.69-1.18 | | 0.92,0.73-1.14 | 1.28,1.00-1.63 |
| Heavy alcohol drinking |  | 1.27(1.01-1.60) | | 0.91(0.71-1.16) | 1.11,0.89-1.37 |
| Unhealthy diet |  | 1.29,1.07-1.55 | | 1.55,1.29-1.88 | 1.60,1.34-1.91 |
| Inactive exercise |  | 1.02.0.85-1.24 | | 1.20,1.00-1.44 | 1.33,1.09-1.63 |
| BMI≥24Kg/m^2^ |  | 1.01,0.83-1.23 | | 2.61,2.15-3.18 | 2.64,2.21-3.17 |
| **Model 3** |  |  | |  |  |
| Smoking |  | 0.92,0.70-1.20 | | 0.87,0.46-1.58 | 1.28,1.00-1.64 |
| Heavy alcohol drinking |  | 1.26.1.00-1.58 | | 0.91,0.71-1.17 | 1.13,0.92-1.40 |
| Unhealthy diet |  | 1.30,1.08-1.57 | | 1.55,1.28-1.87 | 1.57,1.32-1.88 |
| Inactive exercise |  | 1.00,0.82-1.21 | | 1.15,0.95-1.38 | 1.30,1.06-1.59 |
| BMI≥24Kg/m^2^ |  | 1.00,0.82-1.22 | | 2.57,2.11-3.13 | 2.58,2.15-3.10 |
| **Model 4** |  |  | |  |  |
| Smoking |  | 0.88,0.67-1.15 | | 0.86,0.69-2.15 | 1.29,1.00-1.66 |
| Heavy alcohol drinking |  | 1.15,0.91-1.46 | | 0.90,0.71-1.16 | 1.06,0.85-1.33 |
| Unhealthy diet |  | 1.18,0.98-1.43 | | 1.49,1.23-1.80 | 1.52,1.27-1.82 |
| Inactive exercise |  | 0.99,0.82-1.21 | | 1.16,0.96-1.40 | 1.29,1.06-1.59 |
| BMI≥24Kg/m^2^ |  | 0.84,0.68-1.03 | | 2.38,1.95-2.90 | 2.16,1.79-2.61 |

* The reference group was those without the unhealthy lifestyles factor.

**Table S4.** Associations of unhealthy lifestyles with HTH based on 4012 participants: subgroup analyses*.

| **Subgroups** |  | **Odd Ratio** |  | **95%CI** |  | ***p value*** |
| --- | --- | --- | --- | --- | --- | --- |
| Female |  |  |  |  |  |  |
| 0 |  | 1.00 |  |  |  |  |
| 1 |  | 0.71 |  | 0.23-2.23 |  | 0.556 |
| 2 |  | 0.47 |  | 0.16-1.42 |  | 0.179 |
| 3 |  | 0.97 |  | 0.33-2.88 |  | 0.956 |
| 4 |  | 1.46 |  | 0.49-4.39 |  | 0.496 |
| 5 |  | 2.34 |  | 0.73-7.51 |  | 0.155 |
| Male |  |  |  |  |  |  |
| 0 |  | 1.00 |  |  |  |  |
| 1 |  | 1.33 |  | 0.70-2.54 |  | 0.385 |
| 2 |  | 1.41 |  | 0.75-2.66 |  | 0.281 |
| 3 |  | 2.28 |  | 0.62-8.38 |  | 0.214 |
| 4 |  | 3.09 |  | 0.64-34.46 |  | 0.129 |
| 5 |  | 4.69 |  | 1.60-8.38 |  | 0.001 |
| 45 years or older |  |  |  |  |  |  |
| 0 |  | 1.00 |  |  |  |  |
| 1 |  | 1.12 |  | 0.68-2.17 |  | 0.512 |
| 2 |  | 1.07 |  | 0.61-1.88 |  | 0.809 |
| 3 |  | 2.18 |  | 1.23-3.87 |  | 0.008 |
| 4 |  | 3.02 |  | 1.60-5.70 |  | 0.001 |
| 5 |  | 4.86 |  | 2.30-10.30 |  | <0.001 |
| Less than 45 years |  |  |  |  |  |  |
| 0 |  | 1.00 |  |  |  |  |
| 1 |  | 0.53 |  | 0.08-3.51 |  | 0.508 |
| 2 |  | 1.95 |  | 0.39-9.59 |  | 0.413 |
| 3 |  | 2.35 |  | 0.42-13.20 |  | 0.330 |
| 4 |  | 5.01 |  | 0.86-29.05 |  | 0.073 |
| 5 |  | 5.04 |  | 0.64-39.57 |  | 0.124 |
| Prevalent comorbidities |  |  |  |  |  |  |
| 0 |  | 1.00 |  |  |  |  |
| 1 |  | 1.15 |  | 0.64-2.08 |  | 0.639 |
| 2 |  | 1.36 |  | 0.77-2.40 |  | 0.286 |
| 3 |  | 2.52 |  | 1.41-4.52 |  | 0.002 |
| 4 |  | 3.57 |  | 1.89-6.73 |  | <0.001 |
| 5 |  | 6.81 |  | 3.19-14.57 |  | <0.001 |
| Non-prevalent comorbidities |  |  |  |  |  |  |
| 0 |  | 1.00 |  |  |  |  |
| 1 |  | 0.62 |  | 0.13-2.93 |  | 0.541 |
| 2 |  | 0.44 |  | 0.10-2.09 |  | 0.300 |
| 3 |  | 0.96 |  | 0.20-4.56 |  | 0.956 |
| 4 |  | 1.96 |  | 0.36-10.79 |  | 0.439 |
| 5 |  | 2.58 |  | 0.89-4.19 |  | 0.297 |

* All model were adjusted for age, sex, education, family income, marital status, occupational status, history of prevalent comorbidities (including ischemic heart disease, stroke and diabetes), FPG, TC, TG, LDL-C and HDL-C and CRP.


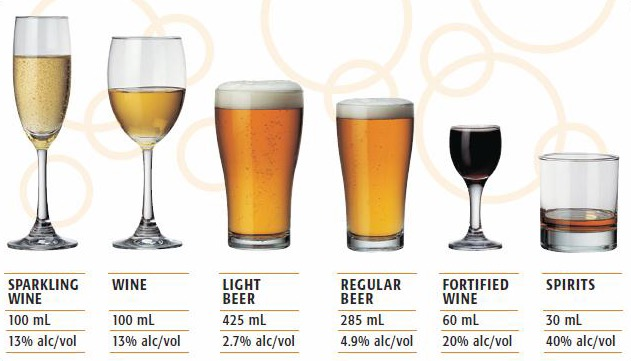


**Figure S1**. Photos to measure the drinks of different kinds of alcohol drinking.
